# Valorization of Cellulosic Waste from Artichoke for Incorporation into Biodegradable Polylactic Acid Matrices

**DOI:** 10.3390/polym16192778

**Published:** 2024-09-30

**Authors:** Alexandra Llidó Barragán, Alejandro de la Calle Salas, Francisco Parres García, José Enrique Crespo Amorós

**Affiliations:** Department of Mechanical and Materials Engineering, Universitat Politècnica de València (UPV), Plz. Ferrándiz y Carbonell, s/n, 03801 Alcoy, Spain; aldel14a@epsa.upv.es (A.d.l.C.S.); fraparga@dimm.upv.es (F.P.G.); jocream@dimm.upv.es (J.E.C.A.)

**Keywords:** circular economy, artichoke, PLA, revaluation

## Abstract

This study presents the development of ecological compounds using polylactic acid (PLA) and artichoke flour with the aim of obtaining materials with properties like commercial PLA. PLA biocomposites with different concentrations of green artichoke (HV) and boiled artichoke (HH) (1, 3, 5, 7, 10 and 20% by weight) were manufactured through an extrusion and injection process. Structural, mechanical, physical and color tests were carried out to analyze the effect of lignocellulosic particles on the biopolymeric matrix. The Shore D hardness, elongation at break and heat deflection temperature (HDT) of the PLA/HV and PLA/HH samples showed similar values to pure PLA, indicating that high concentrations of both fillers did not severely compromise these properties. However, reductions in the tensile strength, impact strength and Young’s modulus were observed, and both flours had increased water absorption capacity. FTIR analysis identified the characteristic peaks of the biocomposites and the ratio of the groups regarding the amount of added filler. The SEM revealed low interfacial adhesion between the polymer matrix and the filler. This study represents a significant advance in the valorization and application of circular economy principles to agricultural waste, such as artichoke waste. PLA/HV biocomposites make a substantial contribution to sustainable materials technology, aligning with the goals of the 2030 agenda to reduce environmental impacts and promote sustainable development.

## 1. Introduction

Artichoke, scientifically known as Cynara cardunculus var. Scolymus, has gained great relevance in agriculture in regions with temperate climates, with Spain being one of the main producing countries, producing 185.6 tons in 2023. Regarding the management of by-products, 60% of by-products from the total production process of artichoke is destined for the canning industry, while the remaining 40% is consumed fresh [1]. In the artichoke industry, the inner bracts are used along with the artichoke receptacle, while the leaves and stems are discarded. These wastes, which represent approximately 70% by weight of the artichoke flower, have been subject to transformation, revaluation and/or elimination. The aim of this study is to revalue these wastes, which contain a significant amount of crude fiber composed of cellulose (65%), hemicellulose (21%) and lignin (14%) [1]. Artichoke residues, especially the leaves, have a remarkable concentration of the enzyme ascorbate peroxidase (APX) along with kinarin and other phenolic compounds. These plant fibers are made up of cellulose, lignin, hemicellulose and pectin, each of which plays a crucial role in their structural properties.

Cellulose, for example, significantly influences the macroscopic characteristics of plant fibers, increasing their tensile strength and Young’s modulus; this results in greater structural rigidity and stability. Lignin, on the other hand, provides an effective defense against attacks from microorganisms, contributing to the durability of the fibers. Pectin is responsible for giving flexibility to the fibers, allowing them to maintain some elasticity. In addition, these two components act as adhesive agents [2]. In contrast, a higher hemicellulose content improves the moisture absorption capacity and accelerates the biodegradation process, thus facilitating faster and more efficient decomposition [3].

This knowledge of the properties of artichoke residues is essential in industry, where they are used in the production of polymer matrix compounds. By taking advantage of these characteristics, the strength and flexibility of the products are demonstrated, contributing to the reduction in waste and the development of more sustainable solutions.

The widespread use of plastics in various sectors has also given rise to concerns about their negative environmental impact, reaching a global figure of 400.3 million tons in 2022 with projections that it will continue growing. To address this problem, the exploration of more sustainable alternatives such as biopolymers derived from renewable and/or biodegradable sources has increased [4,5,6]. In response to growing environmental awareness, polylactic acid (PLA) has emerged as a biodegradable alternative to conventional plastics [7,8,9]. PLA is a synthetic thermoplastic polymer derived from renewable sources, such as corn starch or sugar cane, and it comes from the lactic acid generated by the iron–anaerobic digestion of carbonated materials [10]. The characteristics of commercial PLA are a tensile strength of 32.22 MPa, an elongation at break of 30.7% and a glass transition temperature (Tg) of 63.8 °C [11]. However, the physical properties and biodegradability of PLA depend on its stereochemistry and molecular weight. In the case of L-lactide, a semi-crystalline crystallinity of 37% is obtained, and it is a transparent and hard polymer which has a tensile strength of 4570 MPa and an elongation at break ranging from 85 to 105%. Between the thermal characteristics, it has a vitreous transition (Tg) of 53 °C and a melting point (Tm) of 170–180 °C [12,13]. PLA (DL-Lactida) is considered an amorphous polymer that has no melting point and achieves a glass transition (Tg) of 55 °C. Also, it has a low tensile strength [11,14]. However, PLA has drawbacks such as low thermal stability and brittleness [15]. In addition, its rapid degradation into the environment makes it more sustainable than conventional plastics. It is used in a wide range of applications, from packaging to the construction sector, due to its properties and renewable origin. Research continues to focus on improving the properties of PLA with lignocellulosic reinforcements, thus contributing to reducing the environmental impact of plastics in the future. For example, in a previous study, lignocellulose-rich biowaste with a booster charge (pecan nut (PNS)) and chemically modified polylactic acid were used, revealing an improvement in the behavior of PLA regarding biodegradation [16]. Another example is the work carried out by several authors on the biodegradation process of PLA and TPS, which are compounds used in the manufacture of packaging, in which the stages of biodegrading are detailed. The main features of this process are disintegrating, fragmentation and mineralization [17]. In addition, another study investigated the swelling and biodegradability properties of a biocomposite based on low-molecular-weight polylactic acid (ELP) combined with wheat straw and wood sawdust with the aim of using it as a soil conditioner. The results show that the incorporation of lignocellulosic material into the ELP improved the water retention capacity by 10% as well as its stability in the soil environment. Likewise, the rate of swelling of the biocomposite increased to 300% [18]. On the other hand, another recent investigation revealed that the incorporation of chemically modified rice straw improved the thermal and mechanical properties of PLA, such as its tensile strength, Young’s modulus and glass transition temperature (Tg) [19]. Likewise, an additional study demonstrated that it has excellent mechanical properties due to the strong adhesion between the reinforcement of date palm fibers and low-molecular-weight polylactic acid (ELP) [20]. 

To sum up, the main objective of this study is to develop materials for industrial applications based on PLA (polylactic acid) with additives from artichoke waste. Research is carried out on the interaction between two types of waste, green artichoke (HV) and boiled artichoke (HH), with a PLA matrix in injection-molded tensile samples with different weight contents. The obtained materials were characterized mechanically by tensile tests. In addition, a thermal analysis (TGA) and a structural analysis (FTIR) were performed. The morphology of the fracture surfaces was analyzed using scanning electron microscopy (SEM). Colorimetry and water absorption tests were also carried out.

## 2. Experimental

### 2.1. Materials

In this study, we used polylactic acid (PLA) RXP 7053 NATURAL (PLA/9/1000μ), manufactured by NatureWorks (Plymouth, MN, USA) and supplied by the Resinex Group (Tarragona, Spain). This commercial grade is characterized by being a completely amorphous polymer with a glass transition (Tg) of 55 °C that is obtained by polymerization of the racemic mixture D, L-LA [21] with a melt flow rate of 0.9 g/min (210 °C) and a density of 1.24 g/cm^3^ [22]. Artichoke, provide by Conservas El Raal, S.L.U (Murcia, Spain), had a cellulose content of 21 wt.%, a hemicellulose content of 21 wt.% and a lignin content of 14 wt.% This residue was provided in green (HV) and boiled (HH) states.

#### 2.1.1. Sample Preparation

First, the leaves of both types of artichoke were dried at 50 °C for 72 h in an oven to remove moisture. The leaves were then ground to obtain HV and HH powders.

After the processing, PLA powders and artichokes were manually pre-mixed in different containers according to the formulations described in Table 1 and left to dry in an oven at 50 °C for 12 h to prevent the hydrolysis of PLA. The extrusion process followed by injection molding was performed on an Xplore MC 15HT microcomponent and an Xplore IM12 microinjection molding machine, which were both supplied by Xplore Instruments BV (Sittard, The Netherlands). The temperature profile for the extrusion process was 145–160–155 °C with a speed of 100 rpm. The mixture was kept in the chamber for 1 min to ensure homogeneity. To produce 1BA type samples, the material mixed in the microinjector was introduced at a temperature of 170 °C (injection nozzle) and 30 °C in the mold with a pressure of 16 bar, and the injection and cooling time was 4 s.

#### 2.1.2. Particle Size Measurement

To obtain the particle sizes, a Microtrac MRB particle analyzer with a Sync particle analyzer (Hann, Germany) was used. This equipment is characterized for analyzing the size and shape of particles from 0.01 to 4000 microns using the laser diffraction ISO 13320:2020 standard and dynamic image analysis ISO 13322-2:2006 standard.

#### 2.1.3. Thermal Properties Measurement

To obtain the mass percentage during the material decomposition stages, the STA 449F5 Jupiter^®^ thermobalance from NETZSCH, (Weimar, Germany) was used. The equipment offers high resolution, a wide temperature range, low balance drift and a highly sensitive DSC signal. The samples were placed in a standard 85 µL alumina (Al_2_O_3_) crucibles with an average weight between 7 and 9 mg. For the tests, mass flow controllers (MFCs) used oxygen and argon at rates of 252.5 mL/min and 249.3 mL/min, respectively. These were subjected to a heating program from 40 to 700 °C with a heating rate of 10 °C/min in air atmosphere.

#### 2.1.4. Mechanical Properties Measurement

The mechanical properties of the samples were evaluated using an ELIB 30 universal electromechanical testing machine manufactured by Ibertest (Madrid, Spain) with a 5 kN load cell. All tests were carried out following the UNE-EN ISO 527 standard at a speed of 5 mm/min.

The impact test used a 6J Charpy pendulum from Metrotec S.A. (San Sebastián, Spain) in accordance with the ISO-179 standard.

To obtain a complete mechanical characterization, the Shore D hardness was measured with the JBA 673-D durometer from J. Bot S.A. (Barcelona, Spain) in the injection-molded samples with dimensions of 80 × 10 × 4 mm according to the ISO 868:2003 standard, applying a force of approximately 20 N, using an indenter with an angle of 30° and a tip radius of R0.1. In this test, measurements were made at five different points of the samples with a stabilization time of 15 s.

The values of all mechanical parameters were calculated as an average over 5 specimens for each composition. All tests were carried out at room temperature.

#### 2.1.5. Electron Microscopy (SEM)

An analysis of the morphology of the fractured surfaces of the PLA/HV and PLA/HH tensile specimens was performed using a ZEISS ULTRA 55 field emission scanning electron microscope (FESEM) from Oxford Instruments (Abingdon, UK), operating at an accelerating voltage of 2 kV. Before observation, samples were coated with a 5–7 nm Au layer under vacuum conditions.

#### 2.1.6. Colorimetry

To measure the color of the samples, a Hunter Diffuse model colorimeter (Hunterlab, Reston, VA, USA) was used. The color indices (L*, a* and b*) were measured according to the following criteria: L* is the luminosity and varies from 0 to 100; a* represents the chromatic variable from green (−a*) to red (+a*), and b* represents the chromatic variable from blue (−b*) to yellow (+b*). In the test, 3 measurements were taken, determining their average. The total color difference (AE) was studied using expression (1):

(1) $$\mathrm{AE}=\sqrt{{\left(\Delta L^{*}\right)}^{2}+{\left(\Delta a^{*}\right)}^{2}+{\left(\Delta b^{*}\right)}^{2}}$$

where ∆L*, ∆a*, and ∆b* are the differences in color parameters between the samples and the control film (L* = 30.9; a* = −0.4; b* = 3.8).

#### 2.1.7. Water Uptake Characterization

The water absorption capacity was studied using injection-molded samples of 80 × 10 × 4 mm, which were immersed in distilled water at a temperature of 23.1 °C. Previously, all samples had been dried at 50 °C for 24 h. Samples were then cooled to room temperature, and the initial weight (Wi) of the samples was measured using the electronic analytical balance AG245 from Mettler Toledo Inc. (Schwerzenbach, Switzerland). After obtaining the initial weights, samples were immersed during the established time, and each time, the surface water of the samples was removed with a paper. This process was repeated for a total of 7 weeks with weight change assessments performed every 24 h during the first 3 days and every 7 days for the rest.

#### 2.1.8. FTIR Analysis

Attenuated total reflectance–Fourier transform infrared spectroscopy (FTIR-ATR) was carried out using a Perkin Elmer Spectrum Two FT-IR spectrometer (PerkinElmer, MA, USA) equipped with a universal ATR accessory to identify the functional groups of HV and HH and the different samples of PLA/HV and PLA/HH blends. For this test, FTIR spectra were recorded with a wavenumber range from 4000 to 600 cm^−1^ with a resolution of 4.0 cm^−1^ and an interval of 1.0 cm^−1^.

## 3. Results and Discussion

### 3.1. Particle Size Measurement

**Figure 1:**
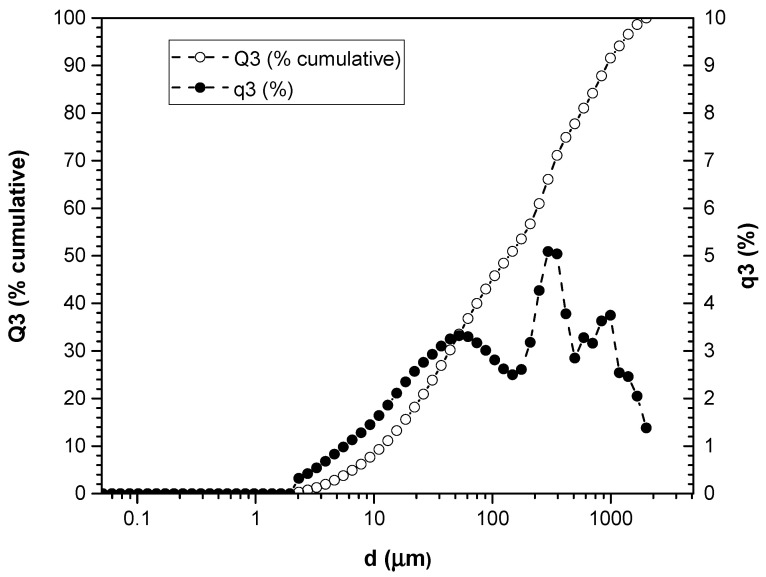
Particle size distribution of HV.

**Figure 2:**
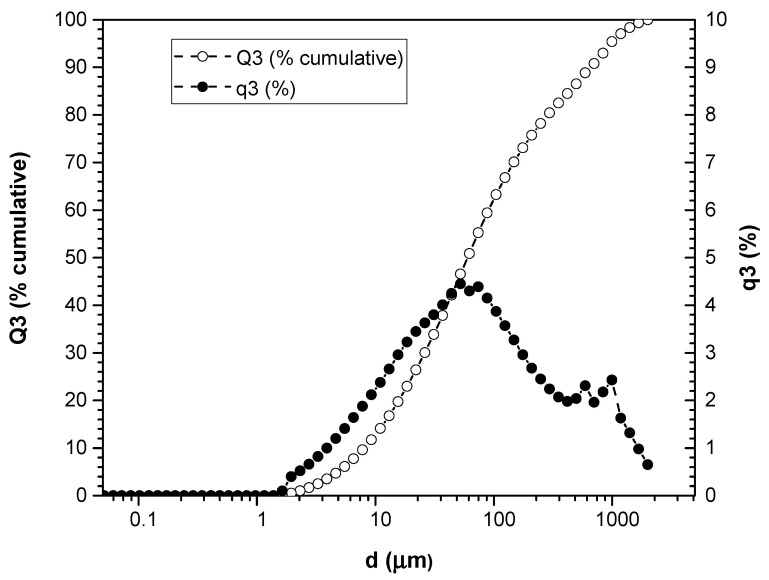
Particle size distribution of HH.

The results of the analysis of the particle size distribution by laser diffraction and dynamic analysis are presented in Figure 1 and Figure 2. These graphs show the size of the particles (μm) on the horizontal axis, while on the vertical axes, both the accumulated percentage of particles larger than or equal to that size in relation to the total particles, called Q3 (%P), and the accumulated percentage of particles smaller or equal to that size in relation to the total particles are represented, which are called q3 (%).

Analysis of the graph in Figure 1 provides a detailed understanding of how the particle size distribution changes in the green (HV) artichoke leaf sample. It is highlighted that the line representing the accumulated percentage of large particles (Q3 (%P)) shows a clear tendency to decrease as the particle size decreases, suggesting a reduction in the accumulation of larger particles in the distribution. For example, between 2000 and 500 μm, this reduction is gradual but constant, while for even smaller particles, this trend appears to be more pronounced. On the other hand, the curve that represents the accumulated percentage of small particles (q3 (%)) follows a different pattern: it is initially low and gradually increases with decreasing particle size. However, it reaches a point where it stabilizes at a lower value. For example, for particles of around 1000 μm, the percentage is relatively low, but as the particles become smaller, it increases significantly until it reaches approximately 3.5%, remaining stable thereafter. An inverse relationship between Q3 (%P) and q3 (%) was observed at certain points on the graph, where while Q3 (%P) decreases, q3 (%) increases.

Analysis of boiled artichoke leaf (HH) shows a clear decrease in the accumulated percentage of large particles as its size decreases, indicating a trend toward a greater accumulation of larger particles in the distribution. On the other hand, the accumulated percentage of smaller particles initially increases with decreasing particle size, although it later stabilizes at low values. This trend suggests a more uniform distribution of small particles. Furthermore, an inversion is observed in the relationship between the cumulative percentages of large and small particles at certain points on the graph, which could indicate changes in the particle size distribution depending on their size.

The analysis of the particle size of artichoke leaves reveals a complex distribution ranging from 2.13 to 2000 μm for HV and from 1.635 to 2000 μm for HH. This difference indicates that leaves subjected to a boiling process are broken down into smaller particles. These differences therefore have significant implications for their application.

### 3.2. Thermal Properties

Thermogravimetric analysis is important to determine the mass percentage of samples. The degradation start temperatures (T_5%_) were obtained when a mass loss of 5% was reached. The maximum decomposition temperatures (T_max_) were calculated from the first derivative of the thermal decomposition rate curves (DTG). The combustion characteristics of HV and HH flour are shown in Table 2.

**Figure 3:**
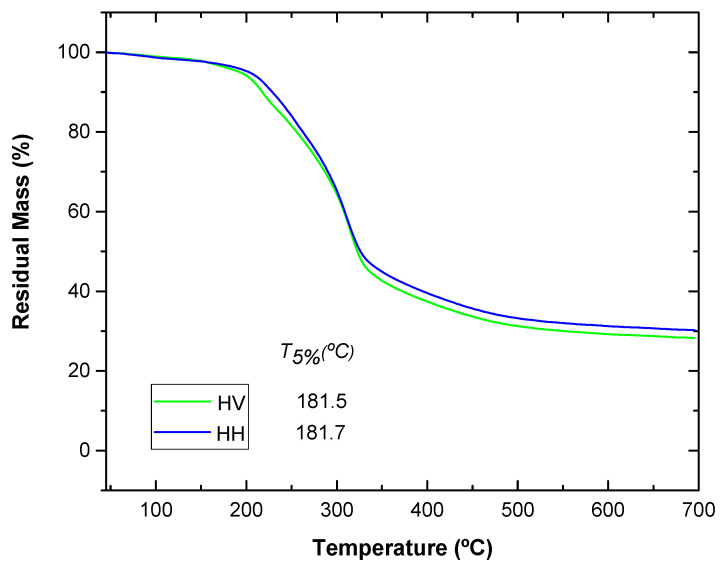
Comparison of TGA curves of HV and HH.

In Figure 3, the different TGA curves for each type of artichoke leaf are presented. It can be observed that the degradation onset (T5%) takes place at almost identical temperatures: 181.5 °C for the leaf in the green state (HV) and 181.7 °C for the boiled leaf (HH) [22,23]. At first glance, a difference is noticeable in the representation of the curves, where the boiled leaf shows a slightly lower percentage of lignin: 11.49% compared to 13.38% for the green leaf [24]. This is due to the process of citric acid addition to the artichoke leaf, which causes changes in the chemical composition, especially in the reduction in lignin content.

**Figure 4:**
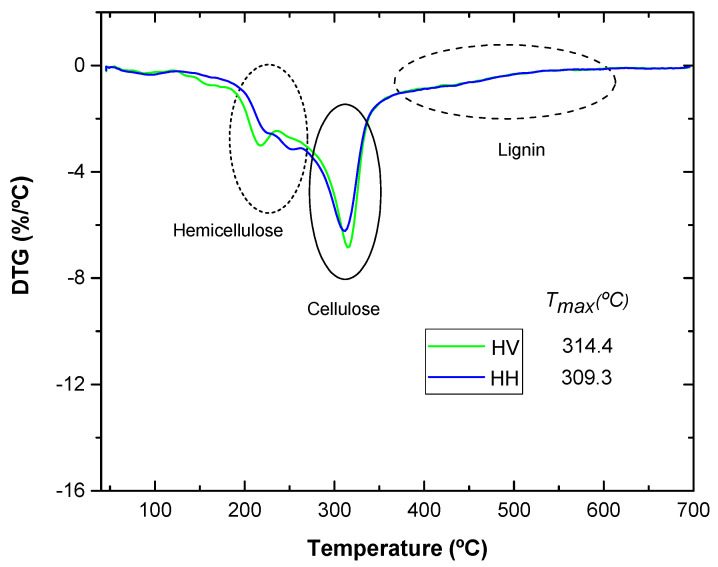
Comparison of DTG curves of HV and HH.

Figure 4 shows a comparison of the thermal decomposition rate (DTG) curves of various artichoke flours. In these curves, the decomposition stages associated with the peaks of hemicellulose, cellulose and lignin can be clearly distinguished. It is notable to highlight the maximum peaks for each type of artichoke flour examined. For example, artichoke leaf flour in the green state reaches a maximum peak of 314.4 °C, while the boiled leaf reaches a maximum peak of 309.3 °C [22,24,25]. The only significant difference is observed in the hemicellulose decomposition stage, where the boiled leaf presents a curve with two peaks due to the decomposition of citric acid at around 250 °C [26].

**Figure 5:**
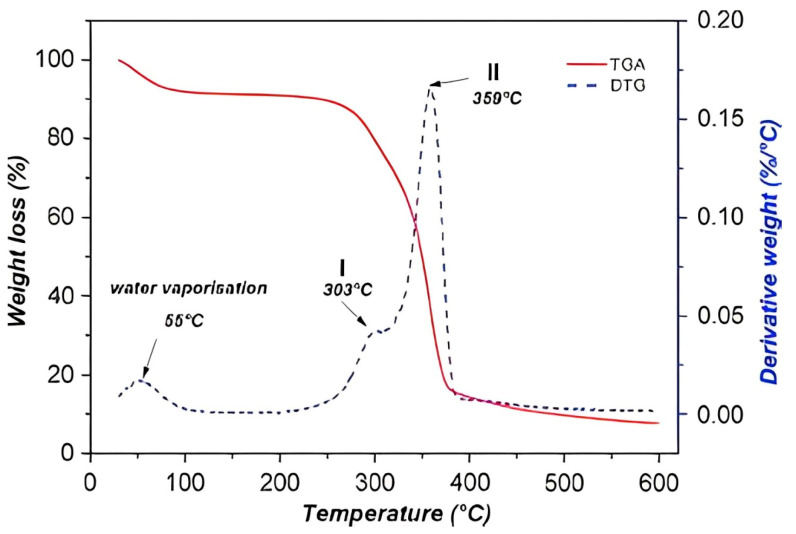
Curve TG and DTG of okra fibers.

The last graph can be related to a previous study that is based on the thermal characterization of okra fibers as a reinforcement of polymeric compounds, in which TG and DTG curves with a similar pattern were obtained [22]. In Figure 5, the degradation onset of okra fibers (220 °C) and their different decomposition stages are shown. The first stage (220–310 °C) causes a weight loss of 16.1%; while the second stage at 310–390 °C produces a weight loss of 60.6% [27] and lignin decomposition (an extended range of temperatures).

In conclusion, in the present project, the thermal analysis curves reveal that the artichoke leaves are stable until around 180 °C in both cases. This is related to the values of natural fibers provided in the literature [28,29].

### 3.3. Mechanical Properties

The mechanical properties of a material are critical in determining its suitability for specific applications. Tensile and impact testing is essential, as it provides vital information on properties such as tensile strength, elongation at break and the ability to withstand impacts. These characteristics are detailed in Table 3.

PLA exhibits brittle behavior, with low elongation capacity but high tensile strength, with typical values of 60.9 MPa for tensile strength, 1134 MPa for Young’s modulus (E) and 7% of elongation at break. The incorporation of HV into PLA clearly showed a significant reduction in the maximum stress as the filler content increased. Several investigations have examined the mechanical properties of natural flours in combination with a polymeric matrix [23]. One of these studies by Ki Wook Kim reveals that the tensile strength of PLA in biocomposites decreases as the amount of added cassava and pineapple flour increases [30]. In the current project, the incorporation of 20% by weight of artichoke in the green state decreased by 29.06%, and it decreased by 33.33% in the boiled state. This phenomenon could be attributed to the milling method applied, which affects the grain size of the different artichokes as well as their physical and morphological characteristics [31]. Likewise, a trend is observed toward a reduction in the elongation capacity with the incorporation of these flours up to 20%. As for the Young’s modulus, in the case of HV, the stiffness increases up to a maximum of 1201.8 MPa at 7% by weight and decreases when raising the added load to a minimum value of 1120 MPa. On the other hand, in the case of HH, a slight increase in the Young’s modulus is observed with the addition of 1 and 3% by weight; however, at higher load values, like 20%, Young’s modulus values are obtained below that of the polymer matrix (1133.7 MPa). This makes sense, because by adding a charge to a polymer, its elasticity and ductility are reduced. Instead of acting as a reinforcement, the load mechanically weakens the sample, decreasing the tensile modulus. At higher concentrations, the particles cluster together, creating weak points and negatively affecting the stiffness of the material [32]. On the other hand, the differences in HV and HH are due to the boiling process that could alter the structure of the leaves, affecting how they disperse.

Among other mechanical characteristics are the hardness and impact strength. In relation to hardness, a slight increase is observed with the addition of the filler, which may be related to the reinforcing effect caused by the hard filler in the polymer matrix. Specifically, the initial hardness value of PLA is 77, and an increase of 4.41% in HV and 4.68% in HH has been experienced.

Finally, the impact strength values of pure PLA are between 28 and 34 kJ/m^2^, which are values close to the one in this work, which was 26 kJ/m^2^. The incorporation of this filler caused a notable decrease in the Charpy impact strength, which was initially 25.6 kJ/m^2^ in the PLA, being reduced to values between 11.5 and 13.1 in the developed biocomposites. This reduction represents a decrease of 44.92% in HV and 51.17% in HH. This effect can be attributed to the high artichoke content (20% by weight), which possibly causes high tensile stresses and limited deformation along the sample [33]. As a result, a reduced ability to absorb impact energy is observed.

### 3.4. Morphology

**Figure 6:**
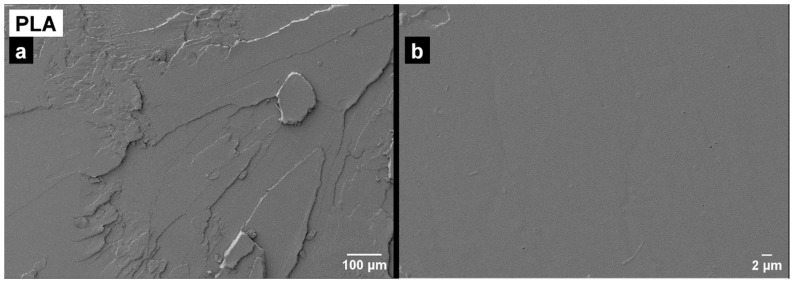
Field emission scanning electron microscopy (FESEM) images (**a**) at 100× and (**b**) at 1500× of the fracture surfaces of the PLA.

Microscopic analysis is crucial to examine the failure surface of materials and understand their mechanical behavior. The image (Figure 6) of the PLA fracture is characterized by the presence of large areas of smooth appearance located at different planes of little unevenness, which is very typical of fragile breaks. Several studies support the low fracture toughness of the polymer [34].

**Figure 7:**
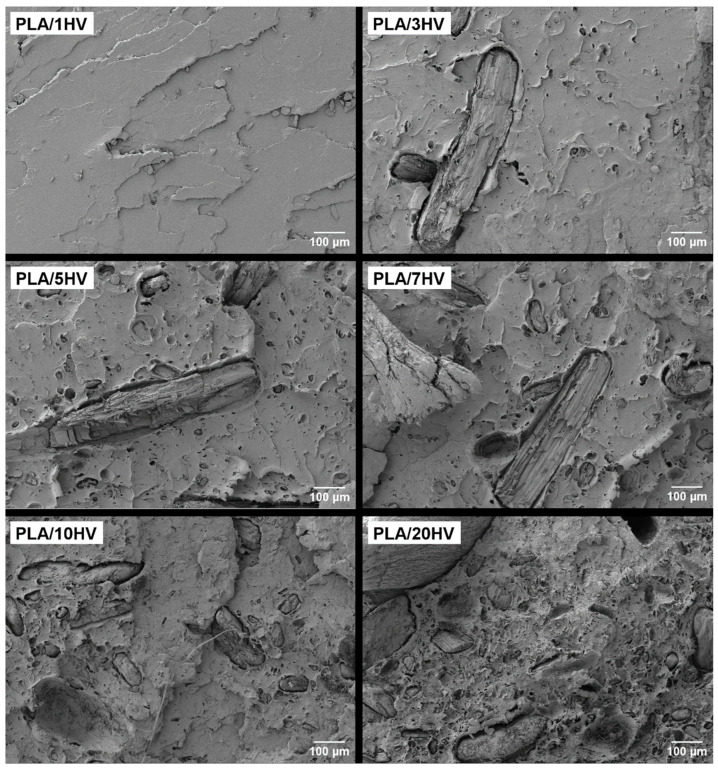
Field emission scanning electron microscopy (FESEM) images at 100× of the fracture surfaces of the PLA/HV composites.

**Figure 8:**
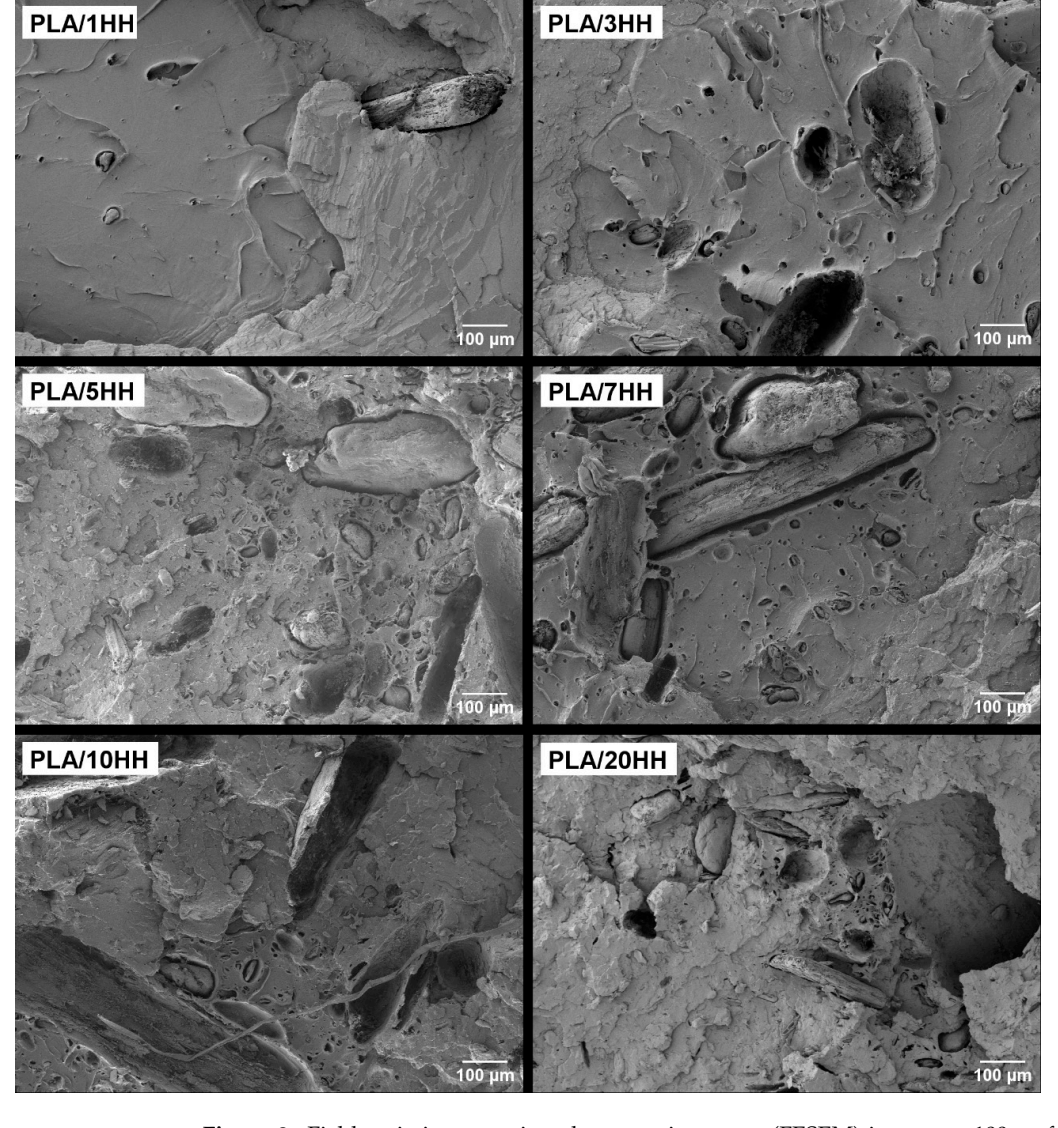
Field emission scanning electron microscopy (FESEM) images at 100× of the fracture surfaces of the PLA/HH composites.

Figure 7 and Figure 8 show that the incorporation of artichoke particles, both green and boiled, causes a substantial change in the breaking surface as the particle percentage increases, being more abrupt as the particle percentage increases. This is a consequence of the presence of cavity particles generated by their removal. In addition, larger particles can be visualized. It is in this type of particles where the lack of adhesion between the matrix phase and the particles can be observed [35]. This phenomenon is seen in both green and boiled particles.

**Figure 9:**
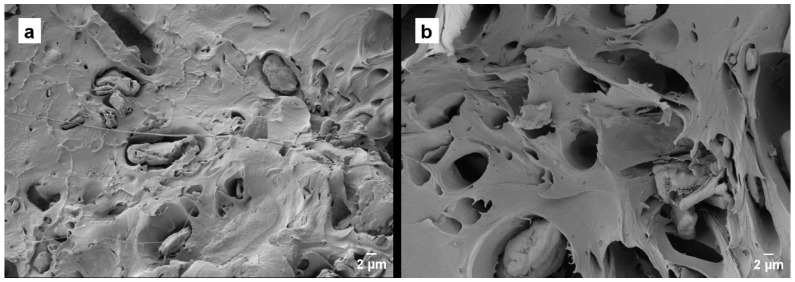
Comparative between field emission scanning electron microscopy (FESEM) images at 1500× of the comparison between composites with the 20% HV and HH. (**a**) PLA/20HV; (**b**) PLA/20HH.

Fractured surfaces of the PLA mixtures with the addition of 20% of HV and HH are contrasted in Figure 9. In Figure 9a, a certain number of incrustations and voids generated by the material can be seen, as well as artichoke particles of various sizes, ranging from 2 to 10 μm. On the other hand, Figure 9b shows a torn surface with depressions, where holes are larger.

The results obtained show that the PLA/HV biocomposites exhibit a clearer surface with more dispersed particles, while in the PLA/HH biocomposites, a greater presence of voids is easily seen. This fact reinforces the initial need to introduce a compatibilization agent in these mixtures to strengthen the adhesion between the PLA and the lignocellulosic filler of the different artichokes.

### 3.5. Color Tests

**Figure 10:**
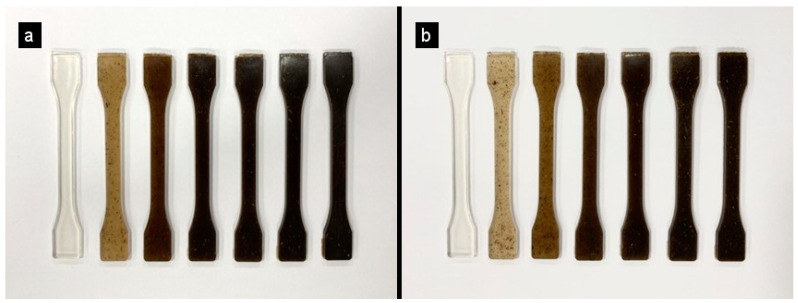
The visible physical appearance of the samples: (**a**) PLA and PLAHV; (**b**) PLA and PLA/HH.

Figure 10 shows the visual appearance of pure PLA and PLA mixtures with both artichokes, HV and HH, which resembles the color of wood. The brown tone has been induced by the injection method due to the temperature reached to inject the PLA.

In addition to the previous qualitative evaluation, color parameters have been studied using the CIELab* system and the total color difference (ΔE) found in Table 4. A marked difference is observed between the polylactic acid samples with and without fillers. Initially, the L* value for PLA is 30, which is considered average due to its natural transparency. With the increase in the loading percentage, the samples acquire a darker tone, going from a light brown tone to a darker one, which translates into a reduction in the luminance value (L*). Other investigations have confirmed these results, demonstrating that the brown color intensifies with the incorporation of more filler to the matrix [36]. Furthermore, the values of the parameters a* and b* are positive, indicating the presence of red and yellow tones, respectively. It is important to note that at the beginning, the different flours had visibly different colors: one was dark brown (HV), and the other was lighter with a yellowish tone (HH). When combined with the transparency of PLA, a similar color was created. This darker tone is confirmed by the L* values obtained for the loaded compounds, which vary between 27 and 29 for a 10% artichoke load.

### 3.6. Water Absorption

Polylactic acid is characterized for being a naturally hydrophobic material that could negatively affect water absorption. For example, several studies have been carried out on the water absorption characteristics of different biomaterials. In particular, the water absorption capacity of a Kenaf core has been examined as an animal bedding material and has shown promising results comparable to commercial materials such as straw and wood chips [37]. Cellulose, which is the most abundant material, shows a strong affinity for water due to the presence of numerous hydroxyl groups [38].

In an additional study, the use of oat husks as a bioabsorbent for natural gas dehydration was investigated, demonstrating a higher water absorption capacity than commercial adsorbents. The oat husk showed a water absorption capacity of 0.63 (g/g), while for molecular sieves, alumina and silica gel values were recorded in the ranges of 0.21–0.26, 0.25–0.33 and 0.35–0.5, respectively [39].

In this case, different artichoke flours were incorporated into the PLA to improve the hydrophobic nature of PLA and take advantage of the management of agricultural waste from artichokes. This by-product contains a high proportion of lignin, cellulose and hemicellulose.

**Figure 11:**
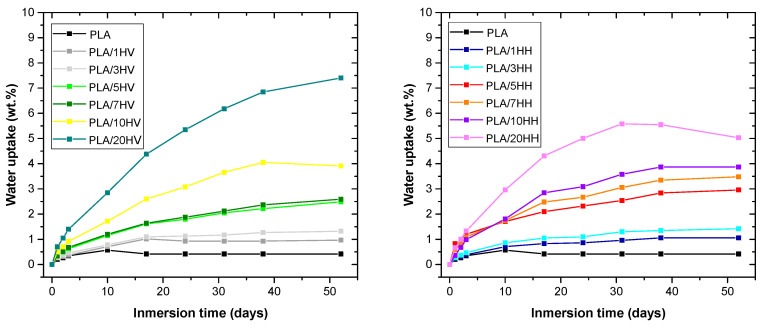
Water uptake of PLA/HV and PLA/HH composites.

Figure 11 illustrates the evolution of the water absorption capacity of the samples manufactured during 52 days of water immersion.

**Figure 12:**
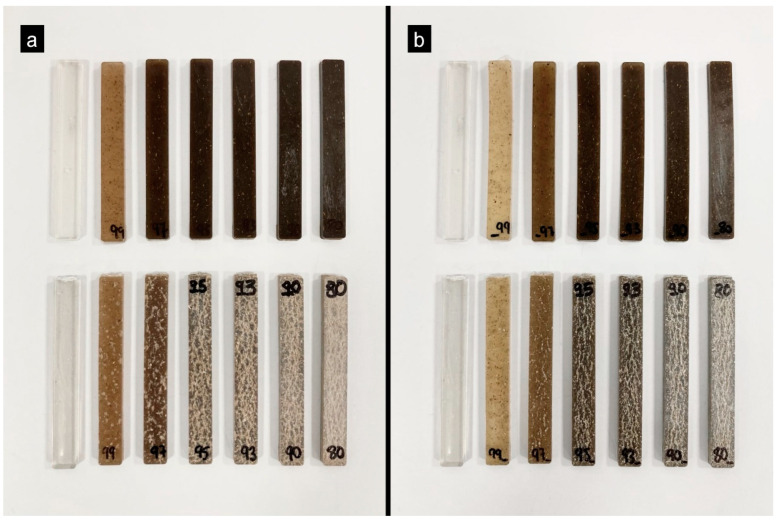
Test pieces obtained after immersion in water. (**a**) PLA/HV composites; (**b**) PLA/HH composites.

It is evident that pure PLA experienced minimal water absorption, recording a value of 0.05% by weight. This can be attributed to the low affinity that PLA has with water, since it is a hydrophobic polymer. By adding the artichoke filler (HV) to the polymer matrix, the water absorption capacity of the biocomposites increases. Artichoke samples of 1%, 3%, 5% and 7% by weight recorded maximum absorption values of between 1% and 3.5% after 52 days. A 10% of HV and HH loading reached values close to 4% by weight, while the 20% sample increased the maximum water absorption capacity to values of 7.4% and 5.03% by weight. This increase is due to the artichoke’s rich composition of cellulose, hemicellulose, pectin and lignin, which can bind functional groups (hydroxyls) with water molecules. However, the difference in water absorption values between HV and HH is because the HH load contains less hemicellulose, lignin and pectin due to the boiling process with citric acid. In summary, the increase in water absorption increases with the percentage of load per age and is most noticeable for HV. However, the incorporation of this residue could improve the water absorption capacity of the polymeric matrix. There was also a gradual degradation of PLA as the percentage of lignocellulosic fillers increased, as shown in Figure 12, which is considered beneficial to accelerate the degradation process of PLA. In addition, color changes and an increased porosity of the material are observed, which facilitates water absorption through the generated pores.

### 3.7. FTIR

**Figure 13:**
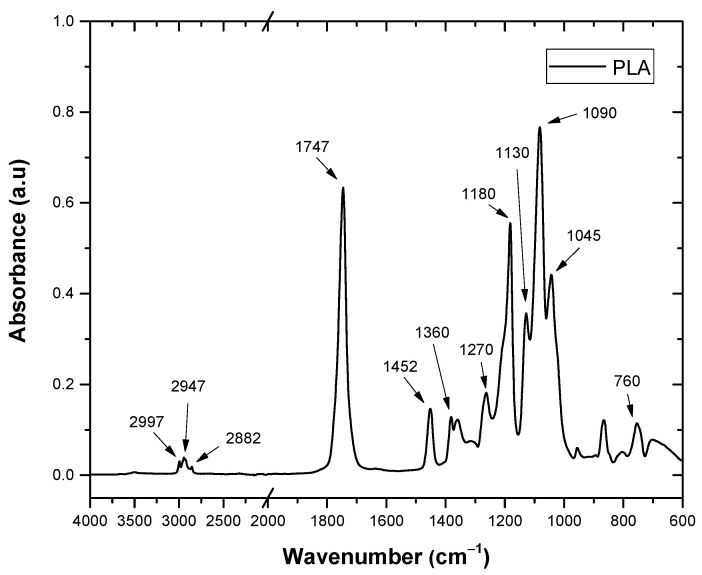
Fourier transform infrared spectrum of the PLA.

The FTIR spectra of PLA, HV and HH chemical function assignments for each absorption band are listed in Table 5. Figure 13 shows the most relevant peaks of PLA. The absorption bands at 1747 cm^−1^ and 1090 cm^−1^ are related to the C=O and C-O groups, respectively. The peaks at 1452 cm^−1^ and 1360 cm^−1^ correspond to the characteristic bands of the symmetric and asymmetric vibrations of the groups [40].

**Figure 14:**
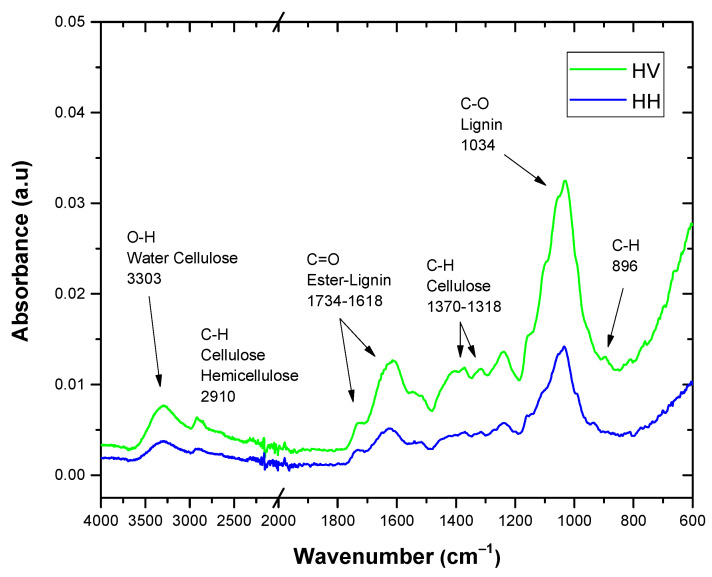
Fourier transform infrared spectrum of the HV and HH.

The FTIR spectra of the HV and HH materials are shown in Figure 14. Several significant peaks stand out: at 3303 cm^−1^, corresponding to the stretching vibration of the O-H group and the hydrogen bonding of the hydroxyl groups [41]; at 2910 cm^−1^, representing the C-H vibration of the aliphatic groups present in cellulose and hemicellulose [42]; at 1734 cm^−1^, associated with the stretching of the carbonyl group of the ester groups [41]; and at 1608 cm^−1^, related to the aromatic benzene of lignin [3]. Furthermore, peaks are observed at 1370 cm^−1^ and 1318 cm^−1^, which are linked to the bending of the C-H and C-O groups of the aromatic ring in the polysaccharides, respectively [43]. The band at 1239 cm^−1^ is attributed to the stretching of the C-O group from the acetyl group of lignin [44]. On the other hand, the intense band centered at 1034 cm^−1^ can be associated with the stretching bond of C-O and O-H belonging to the ether and hydroxyl groups in the polysaccharides, while the peak at 896 cm^−1^ is attributed to the presence of β-glucosidics bonds in monosaccharides [22].

Observing these FTIR spectra, the height of the peaks in the spectrum of the boiled leaf is lower compared to that of the green leaf. This is due to the treatment of the boiled leaf with citric acid (C6H8O7), where similarities and differences between the characteristic bands are identified. Among these similarities and differences are the functional groups of hydroxyl groups (O-H) and carbonyl groups (C=O) [45].

**Figure 15:**
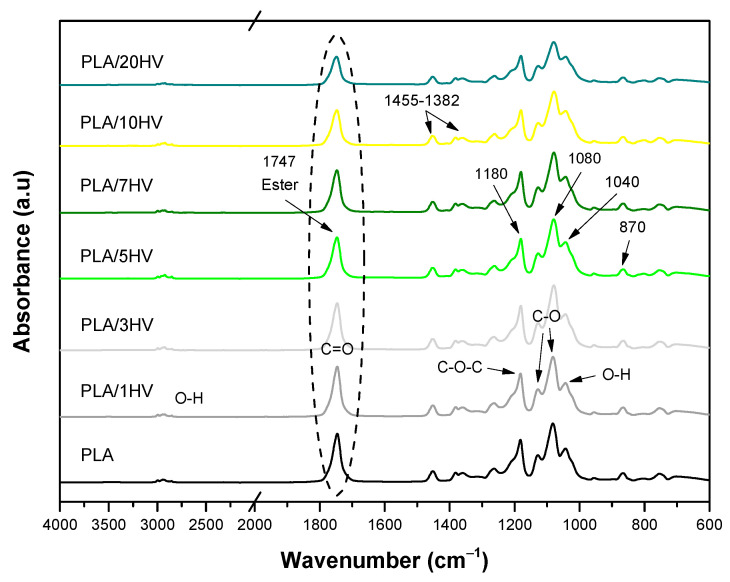
FTIR spectra of PLA formulations with HV.

**Figure 16:**
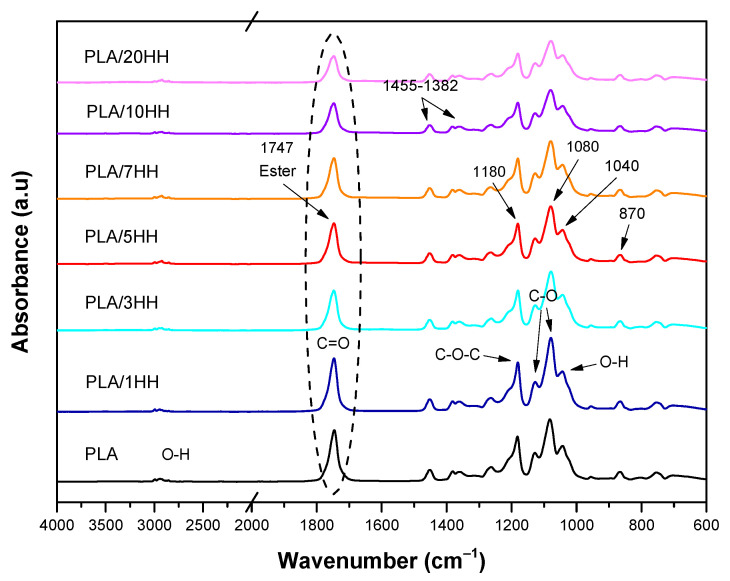
FTIR spectra of PLA formulations with HH.

Figure 15 and Figure 16 show the FTIR spectra, like those of pure PLA, with the addition of HV and HH to the PLA matrix. In the range of 3500 to 3000 cm^−1^, the O-H functional group was identified, which is possibly related to water absorption due to its hydrophilic nature. This peak was present in all samples but disappeared in pure PLA. Furthermore, stretching absorption peaks of C=O, which are associated with the interaction between the carbon groups of PLA and the ester groups of HV and HH, were observed at 1747 cm^−1^, which is in agreement with previous research [43]. Two bands of asymmetric and symmetric bending of the methyl group (CH_3_) were also detected at 1455 and 1382 cm^−1^, respectively. Furthermore, an increase in the bands at 1129 and 1080 cm ^−1^ was noted, which was possibly due to the presence of (C-O), (C=O) groups in hemicellulose and lignin. The O-H stretching vibration was also detected at 1040 cm^−1^, which belongs to polysaccharides in cellulose [46,47]. The difference in the intensity of the peaks between the PLA/HV and PLA/HH samples is related to the content of both artichokes, while the slight shifts between the peaks could be attributed to the interactions between the carbon groups of PLA and the hydroxyl groups of the different artichokes.

## 4. Conclusions

Six different formulations of PLA/HV and PLA/HH mixtures were prepared with contents by weight of 1%, 3%, 5%, 7%, 10% and 20%. TGA revealed the stages of decomposition of each lignocellulosic load, highlighting a greater difference in the stage of decomposition of hemicellulose due to the process of boiling leaves in citric acid. Although these biocomposites showed elongation at break and Shore D hardness values comparable to those of pure PLA, decreases in the tensile strength, Young’s modulus and impact resistance were observed, which were possibly due to low interfacial interaction. The SEM analysis revealed a low adhesion between PLA and artichoke powders, which caused tensile losses, especially in mixtures with HV artichoke powder. The first sample of this type was used in its natural state without further treatment but showed a clearer dispersion of the particles. The FTIR analysis highlighted the influence of the carbonyl group (C=O) and the improvement in leaf dispersion due to ethanol in HV and HH, emphasizing how the use of PLA compounds in plant-based materials, such as artichoke leaves, can affect the properties of materials. The particle size measurement showed that the sample particle size varies between 1.635 and 2000 μm. In the colorimetric test, both artichokes create biocompounds with a brown color degradation, resulting in a visual appearance like wood. The results of the water absorption capacity test indicate that PLA biocompounds with artichoke flour accelerate the degradation process. Despite the mechanical properties of the starting polymer and the antioxidant capacity of the artichoke residue, the process of boiling leaves does not improve the material properties. These results highlight the biocomposite PLA with a 5% load of HV, demonstrating that the HDT-TDF is a highly dynamic system that presents an optimum balance, combining a high load content with minimal losses in mechanical properties such as maximum stress of 7.3% and 6% in HDT. Without any damage, it improves the Young’s modulus by 1.7% and the PLA hardness by 3.3%, in addition to accelerating its degradation with a water absorption of 2.5%. These biocomposites are especially useful in sectors that demand materials with low tensile strength and water degradability, such as biodegradable pots and disposable products. The use of lignocellulosic fillers reduces PLA costs, and the elimination of the boiling process reduces both production costs and time. In conclusion, PLA/5HV biocomposites represent a significant contribution toward sustainable materials, aligning with the 2030 agenda goals for minimizing environmental impacts and promoting sustainable development.

## Figures and Tables

**Table 1:** Summary of compositions according to the weight content (wt.%) of PLA and different proportions of raw artichoke (HV) and boiled artichoke (HH).

Code	PLA (wt.%)	Artichoke (wt.%)
PLA	100	0
PLA/1HV	99	1
PLA/3HV	97	3
PLA/5HV	95	5
PLA/7HV	93	7
PLA/10HV	90	10
PLA/20HV	80	20
PLA/1HH	99	1
PLA/3HH	97	3
PLA/5HH	95	5
PLA/7HH	93	7
PLA/10HH	90	10
PLA/20HH	80	20

**Table 2:** HV and HH combustion characteristics.

Material	Weight Loss (%) in a Temperature Range of 40–180	I Degradation Stage				II Degradation Stage				Lignin Decomposition (% by Weight)
		T_5%_ (°C)	T_90%_ (°C)	T_max_ (°C)	Weight Loss (%)	T_5%_ (°C)	T_90%_ (°C)	T_max_ (°C)	Weight Loss (%)	
HV	5.76	181.5	235.5	217.5	11.49	368.8	235.5	314.4	40.94	13.38
HH	3.50	181.7	265.1	222.7	17.38	373.5	262.7	309.4	37.04	11.49

**Table 3:** Mechanical characteristics of the PLA blends.

Code	τ_max_ (MPa)	ε_b_ (%)	E (MPa)	Shore D Hardness	Impact Strength (kJ/m^2^)
PLA	60.9 ± 2.2	6.9 ± 0.3	1133.7 ± 129.4	77.0 ± 0.5	25.6 ± 5.0
PLA/1HV	59.6 ± 0.55	6.9 ± 0.3	1176.2 ± 58.3	79.0 ± 0.0	20.5 ± 3.1
PLA/3HV	58.2 ± 1.62	7.0 ± 0.9	1141.2 ± 54.2	79.2 ± 0.4	16.4 ± 1.2
PLA/5HV	56.4 ± 0.87	6.3 ± 0.5	1153.2 ± 43.3	79.6 ± 0.5	15.7 ± 0.7
PLA/7HV	55.9 ± 0.76	5.8 ± 0.2	1201.8 ± 7.8	78.8 ± 0.5	14.8 ± 0.4
PLA/10HV	52.8 ± 0.71	5.8 ± 0.3	1120.0 ± 23.2	80.2 ± 0.5	12.1 ± 1.2
PLA/20HV	43.2 ± 2.96	4.4 ± 0.4	1125.4 ± 38.5	80.6 ± 0.9	11.5 ± 0.9
PLA/1HH	57.6 ± 0.6	6.4 ± 0.3	1159.5 ± 36.9	78.6 ± 0.4	19.2 ± 2.9
PLA/3HH	56.3 ± 1.6	6.1 ± 0.3	1172.2 ± 24.6	79.2 ± 0.5	17.4 ± 1.2
PLA/5HH	52.4 ± 0.9	6.3 ± 1.0	1133.9 ± 32.7	79.8 ± 0.0	16.0 ± 2.3
PLA/7HH	50.7 ± 0.8	5.7 ± 0.3	1133.9 ± 34.7	80.0 ± 0.0	15.6 ± 0.7
PLA/10HH	43.9 ± 0.7	5.0 ± 0.2	1085.0 ± 46.7	80.2 ± 0.4	13.5 ± 1.0
PLA/20HH	40.6 ± 2.9	4.5 ± 0.3	1075.7 ± 34.5	80.4 ± 0.5	13.1 ± 1.3

**Table 4:** Optical parameters of pure PLA and the PLA/HV and PLA/HH blends.

Code	L*	a*	b*	ΔE (Control)
PLA	30.9	−0.4	3.8	-
PLA/1HV	25.6	0.4	3.0	5.4
PLA/3HV	24.6	1.5	3.5	6.6
PLA/5HV	25.2	2.4	3.4	6.4
PLA/7HV	25.4	1.7	3.9	5.9
PLA/10HV	26.9	1.9	3.3	4.6
PLA/20HV	29.4	2.4	3.5	3.2
PLA/1HH	25.1	1.9	5.4	6.4
PLA/3HH	27.6	1.7	5.0	4.1
PLA/5HH	27.9	2.1	4.7	4.0
PLA/7HH	27.8	2.1	4.6	4.1
PLA/10HH	28.9	2.6	4.6	3.7
PLA/20HH	28.2	2.9	4.1	4.5

**Table 5:** Characteristic peaks of PLA, HV and HH.

PLA		HV-HH	
Assignment	Wavenumbers (cm^−1^)	Assignment	Wavenumbers (cm^−1^)
CH_3_	2997	OH	3500–3000
-C-CH_3_	2947	CH	2918
-CH, -CH_3_	2882	(Carbonyl, ketone and ester) C=O	1734
C=O	1747	OH hydroxyls	1645
CH_3_	1452	C=O	1618
-CH_3_, CH-CH_3_	1360	CH	1430–1407
COC, -CO	1180	CH, polysaccharides	1370
COC, r_as_ CH_3_	1130	CO	1318
COC	1090	COC of phenol ether bond	1239
CC, COC	1045	CO	1034
CH_3_, C=O	760	CH	896

## Data Availability

The data presented in this study are available on request from the corresponding author due to privacy reasons.

## References

[1] (2012). PINELLI; PATRIZIA. Proceso Para Producir Extractos Nutracéuticos Refinados a Partir de Residuos de Alcachofa y Otras Plantas Del Género Cynara. 2 401 203. ES2401203T3 Patent.

[2] Mohanty A.K., Misra M., Drzal L.T. (2005). Natural Fibers, Biopolymers, and Biocomposites.

[3] Calvo A. (2013). Determinación Experimental y Modelización de Isotermas de Absorción de Agua de Hojas de Alcachofa.

[4] PlasticsEurope. https://plasticseurope.org/wp-content/uploads/2022/10/PE-PLASTICS-THE-FACTS_V7-Tue_19-10-1.pdf.

[5] Malinconico M., Cerruti P., Santagata G., Immirzi B. (2014). Natural polymers and additives in commodity and specialty applications: A challenge for the chemistry of future. Macromol. Symp..

[6] John M.J., Thomas S. (2008). Biofibres and biocomposites. Carbohydr. Polym..

[7] Vilpoux O., Averous L. (2004). Starch-based plastics. Technol. Use Potentialities Lat. Am. Starchy Tubers.

[8] Yu L., Dean K., Li L. (2006). Polymer blends and composites from renewable resources. Prog. Polym. Sci..

[9] Oliveira R.B. (2006). Polímeros na obtenção de sistemas de liberação de fármacos. Rev. Eletrônica De Farmácia.

[10] Anugwom I., Lahtela V., Kallioinen M., Kärki T. (2019). Lignin as a functional additive in a biocomposite: Influence on mechanical properties of polylactic acid composites. Ind. Crops Prod..

[11] Briassoulis D. (2004). Una descripción general del comportamiento mecánico de las películas agrícolas biodegradables. J. Poli. Reinar.

[12] VERT M. (2002). Polymères de fermentation: Les poly (acide lactique) s et leurs précurseurs, les acides lactiques. L’Actualité Chim. (Paris. 1973).

[13] Mochizuki M., Hirami M. (1197). Efectos estructurales sobre la biodegradación de poliésteres alifáticos. Polimero. Adv. Tecnol..

[14] Rutot D., Dubois P. (2004). Les (bio) polymeres biodegradables: L’enjeu de demain?. Chim. Nouv..

[15] Weng Y.X., Jin Y.J., Meng Q.Y., Wang L., Zhang M., Wang Y.Z. (2013). Biodegradation behavior of poly (butylene adipate-co-terephthalate) (PBAT), poly (lactic acid) (PLA), and their blend under soil conditions. Polym. Test..

[16] Sánchez-Acosta D., Rodriguez-Uribe A., Álvarez-Chávez C.R., Mohanty A.K., Misra M., López-Cervantes J., Madera-Santana T.J. (2019). Physicochemical characterization and evaluation of pecan nutshell as biofiller in a matrix of poly (lactic acid). J. Polym. Environ..

[17] Camacho-Muñoz R., Villada-Castillo H.S., Solanilla-Duque J.F. (2020). Anaerobic biodegradation under slurry thermophilic conditions of poly (lactic acid)/starch blend compatibilized by maleic anhydride. Int. J. Biol. Macromol..

[18] Cruz Fabian D.R., Durpekova S., Dusankova M., Cisar J., Drohsler P., Elich O., Borkova M., Cechmankova J., Sedlarik V. (2023). Renewable Poly(Lactic Acid)Lignocellulose Biocomposites for the Enhancement of the Water Retention Capacity of the Soil. Polymers.

[19] Ghorbani Chaboki M., Mohammadi-Rovshandeh J., Hemmati F. (2019). Poly (Lactic Acid)/Thermoplasticized Rice Straw Biocomposites: Effects of Benzylated Lignocellulosic Filler and Nanoclay. Iran. Polym. J..

[20] Ghanmi I., Slimani F., Ghanmi S., Guedri M. (2024). Development and Characterization of a PLA Biocomposite reinforced with Date Palm Fibers. Eng. Technol. Appl. Sci. Res..

[21] Carrión F.J., Avilés M.D., Nakano K., Tadokoro C., Nagamine T., Bermúdez M.D. (2019). Diprotic ammonium palmitate ionic liquid crystal and nanodiamonds in aqueous lubrication. Film thickness and influence of sliding speed. Wear.

[22] De Rosa I.M., Kenny J.M., Puglia D., Santulli C., Sarasini F. (2010). Morphological, Thermal and Mechanical Characterization of Okra (*Abelmoschus Esculentus*) Fibres as Potential Reinforcement in Polymer Composites. Compos. Sci. Technol..

[23] Fiore V., Valenza A., Di Bella G. (2011). Artichoke (*Cynara Cardunculus* L.) Fibres as Potential Reinforcement of Composite Structures. Compos. Sci. Technol..

[24] Poletto M., Ornaghi H.L., Zattera A.J. (2014). Native Cellulose: Structure, Characterization and Thermal Properties. Materials.

[25] Ouajai S., Shanks R.A. (2005). Composition, Structure and Thermal Degradation of Hemp Cellulose after Chemical Treatments. Polym. Degrad. Stab..

[26] Silva N.T., Nascimento N.F., Cividanes L.S., Bertran C.A., Thim G.P. (2008). Kinetics of Cordierite Crystallization from Diphasic Gels. J. Sol-Gel Sci. Technol..

[27] Albano C., Gonzalez J., Ichazo M., Kaiser D. (1999). Thermal stability of blends of polyolefins and sisal fiber. Polym. Degrad. Stab..

[28] Wielage B., Lampke T., Marx G., Nestler K., Starke D. (1999). Thermogravimetric and differential scanning calorimetric analysis of natural fibres and polypropylene. Thermochim. Acta.

[29] Spinacé M.A., Lambert C.S., Fermoselli K.K., De Paoli M.A. (2009). Characterization of lignocellulosic curaua fibres. Carbohydr. Polym..

[30] Kim K.-W., Lee B.-H., Kim H.-J., Sriroth K., Dorgan J.R. (2012). Thermal and Mechanical Properties of Cassava and Pineapple Flours-Filled PLA Bio-Composites. J. Therm. Anal. Calorim..

[31] Taşdemir M. (2017). Effects of Olive Pit and Almond Shell Powder on Polypropylene. Key Eng. Mater..

[32] Quiles-Carrillo L., Montanes N., Jorda-Vilaplana A., Balart R., Torres-Giner S. (2019). A comparative study on the effect of different reactive compatibilizers on injection-molded pieces of bio-based high-density polyethylene/polylactide blends. J. Appl. Polym. Sci..

[33] Morreale M., Liga A., Mistretta M.C., Ascione L., Mantia F.P. (2015). Mechanical, Thermomechanical and Reprocessing Behavior of Green Composites from Biodegradable Polymer and Wood Flour. Materials.

[34] Chaiwutthinan P., Chuayjuljit S., Srasomsub S., Boonmahitthisud A. (2019). Composites of poly (lactic acid)/poly (butylene adipate-co-terephthalate) blend with wood fiber and wollastonite: Physical properties, morphology, and biodegradability. J. Appl. Polym. Sci..

[35] da Silva W.A., Luna C.B.B., de Melo J.B.d.C.A., Araújo E.M., Filho E.A.d.S., Duarte R.N.C. (2021). Feasibility of Manufacturing Disposable Cups Using PLA/PCL Composites Reinforced with Wood Powder. J. Polym. Environ..

[36] Liminana P., Quiles-Carrillo L., Boronat T., Balart R., Montanes N. (2018). The Effect of Varying Almond Shell Flour (ASF) Loading in Composites with Poly (Butylene Succinate (PBS) Matrix Compatibilized with Maleinized Linseed Oil (MLO). Materials.

[37] Lips S.J.J., Iñiguez de Heredia G.M., Op den Kamp R.G.M., van Dam J.E.G. (2009). Water Absorption Characteristics of Kenaf Core to Use as Animal Bedding Material. Ind. Crops Prod..

[38] Al Abdallah H., Abu-Jdayil B., Iqbal M.Z. (2022). Improvement of Mechanical Properties and Water Resistance of Bio-Based Thermal Insulation Material via Silane Treatment. J. Clean. Prod..

[39] Ghanbari S., Niu C.H. (2019). Characteristics of oat hull based biosorbent for natural gas dehydration in a PSA process. J. Nat. Gas Sci. Eng..

[40] Sánchez C. (2023). Desarrollo, Caracterización y Propiedades de Nanofases, Nanofluidos y Nanomaterials. Ph.D. Thesis.

[41] Yang H., Yan R., Chen H., Lee D.H., Zheng C. (2007). Characteristics of Hemicellulose, Cellulose and Lignin Pyrolysis. Fuel.

[42] Paiva M.C., Ammar I., Campos A.R., Cheikh R.B., Cunha A.M. (2007). Alfa Fibres: Mechanical, Morphological and Interfacial Characterization. Compos. Sci. Technol..

[43] Le Troëdec M., Dalmay P., Patapy C., Peyratout C., Smith A., Chotard T. (2011). Mechanical Properties of Hemp-Lime Reinforced Mortars: Influence of the Chemical Treatment of Fibers. J. Compos. Mater..

[44] Liu W., Mohanty A.K., Drzal L.T., Askel P., Misra M. (2004). Effects of alkali treatment on the structure, morphology and thermal properties of native grass fibers as reinforcements for polymer matrix composites. J. Mater. Sci..

[45] Stuart B. (2004). Infrared Spectroscopy: Fundamentals and Applications.

[46] Venkatesh C., Laurenti M., Bandeira M., Lanzagorta E., Lucherini L., Cauda V., Devine D.M. (2020). Biodegradation and Antimicrobial Properties of Zinc Oxide–Polymer Composite Materials for Urinary Stent Applications. Coatings.

[47] Yang S., Wu Z.-H., Yang W., Yang M.-B. (2008). Thermal and Mechanical Properties of Chemical Crosslinked Polylactide (PLA). Polym. Test..

